# Structural basis of tRNA^Pro^ acceptor stem recognition by a bacterial *trans*-editing domain

**DOI:** 10.1093/nar/gkad192

**Published:** 2023-03-23

**Authors:** Xiao Ma, Marina Bakhtina, Irina Shulgina, William A Cantara, Alexandra B Kuzmishin Nagy, Yuki Goto, Hiroaki Suga, Mark P Foster, Karin Musier-Forsyth

**Affiliations:** Department of Chemistry and Biochemistry and Center for RNA Biology, Ohio State University, Columbus, OH 43210, USA; Department of Chemistry and Biochemistry and Center for RNA Biology, Ohio State University, Columbus, OH 43210, USA; Department of Chemistry and Biochemistry and Center for RNA Biology, Ohio State University, Columbus, OH 43210, USA; Department of Chemistry and Biochemistry and Center for RNA Biology, Ohio State University, Columbus, OH 43210, USA; Department of Chemistry and Biochemistry and Center for RNA Biology, Ohio State University, Columbus, OH 43210, USA; Department of Chemistry, Graduate School of Science, University of Tokyo, Bunkyo, Tokyo 113-0033, Japan; Department of Chemistry, Graduate School of Science, University of Tokyo, Bunkyo, Tokyo 113-0033, Japan; Department of Chemistry and Biochemistry and Center for RNA Biology, Ohio State University, Columbus, OH 43210, USA; Department of Chemistry and Biochemistry and Center for RNA Biology, Ohio State University, Columbus, OH 43210, USA

## Abstract

High fidelity tRNA aminoacylation by aminoacyl-tRNA synthetases is essential for cell viability. ProXp-ala is a *trans*-editing protein that is present in all three domains of life and is responsible for hydrolyzing mischarged Ala-tRNA^Pro^ and preventing mistranslation of proline codons. Previous studies have shown that, like bacterial prolyl-tRNA synthetase, *Caulobacter crescentus* ProXp-ala recognizes the unique C1:G72 terminal base pair of the tRNA^Pro^ acceptor stem, helping to ensure deacylation of Ala-tRNA^Pro^ but not Ala-tRNA^Ala^. The structural basis for C1:G72 recognition by ProXp-ala is still unknown and was investigated here. NMR spectroscopy, binding, and activity assays revealed two conserved residues, K50 and R80, that likely interact with the first base pair, stabilizing the initial protein-RNA encounter complex. Modeling studies are consistent with direct interaction between R80 and the major groove of G72. A third key contact between A76 of tRNA^Pro^ and K45 of ProXp-ala was essential for binding and accommodating the CCA-3′ end in the active site. We also demonstrated the essential role that the 2′OH of A76 plays in catalysis. Eukaryotic ProXp-ala proteins recognize the same acceptor stem positions as their bacterial counterparts, albeit with different nucleotide base identities. ProXp-ala is encoded in some human pathogens; thus, these results have the potential to inform new antibiotic drug design.

## INTRODUCTION

In the first step of protein synthesis, aminoacyl-tRNA synthetases (ARSs) are responsible for facilitating accurate interpretation of the genetic code by activating specific amino acids and aminoacylating their corresponding tRNAs (1). In the two-step aminoacylation reaction, cognate amino acids are first activated with ATP to form an aminoacyl-adenylate intermediate. The activated amino acid is next transferred onto its corresponding tRNA to form the covalent aminoacyl-tRNA ester bond. While tRNAs provide a large surface area for accurate discrimination and structural features of ARS catalytic domains provide a sufficient degree of selectivity for accurate activation of some amino acids, errors in aminoacylation can occur, especially for the smaller and isometric amino acids (2). If left uncorrected, mistranslation events may accumulate, generating misfolded or non-functional proteins, with detrimental effects for living cells (3–7). Organisms in all three domains of life encode proofreading or editing domains in about half of their ARSs. These domains are distinct from the synthetic or aminoacylation active site and function to deacylate mischarged tRNA (4). Many organisms also possess single-domain enzymes that function to edit tRNAs *in trans* (7,8).

Based upon structural and functional differences, ARSs are divided into two classes (9). More recently, kinetic differences between the two classes have been described. Whereas the rate-limiting step in the overall aminoacylation reaction is product release for class I synthetases, the chemical step of aminoacyl transfer is rate-limiting for class II ARSs (10). Due to the fast product-release step, *trans*-editing by free-standing editing domains is proposed to be more important for tRNAs charged by class II enzymes (8). Indeed, all naturally-occurring *trans*-editing domains described to date are derived from class II ARS editing domains (8).

Class II prolyl-tRNA synthetase (ProRS) has been shown *in vitro* to misactivate Ala and Cys (11–14). In comparison to other amino acids, mistranslation of Pro codons may be less well tolerated because of its unique ring structure with restricted conformational flexibility (15). Widely varying concentrations of cellular amino acid pools may also increase the demand for aminoacyl-tRNA editing. While the amino acid pool is reported to be fairly stable during different phases of *Escherichia coli (Ec)* cell growth and the levels of Pro remain low throughout, the levels of Ala increase during growth (16). Wild-type (WT) *Ec* cells cultured in a glucose-mineral salt medium are reported to contain 168 μM Ala, 45 μM Cys and only 9 μM Pro in the middle of the exponential growth phase (16). Although *Ec* ProRS discriminates Ala by a factor of 23 000 over cognate Pro *in vitro*, the significantly higher Ala vs Pro concentration in the cell reduces the ‘effective discrimination factor’ to 1200, conditions where mischarging is expected and editing is likely required (11).

The majority of bacterial ProRSs encode an editing domain inserted between the class II consensus motifs 2 and 3, known as the INS domain (17). This domain is responsible for hydrolyzing Ala-tRNA^Pro^ and experiments with an editing-deficient mutant support a model in which ProRS directly transfers the misactivated Ala onto tRNA^Pro^ (11,17). The INS domain fails to edit Cys-tRNA^Pro^ and a ‘triple-sieve’ mechanism was proposed for how bacterial ProRS together with related *trans*-editing domains maintain high fidelity of aminoacylation (18). In the first step, the aminoacylation domain rejects amino acids that are larger than Pro. Next, the INS domain functions as the second sieve to deacylate mischarged Ala-tRNA^Pro^. Finally, a single-domain *trans*-editing factor, YbaK, hydrolyzes mischarged Cys-tRNA^Pro^ as the third sieve (18–20).

For bacteria that encode a ProRS lacking a functional INS domain, such as *Clostridium sticklandii* and *Caulobacter crescentus* (*Cc*), a single-domain enzyme, ProXp-ala, serves as the second sieve (21,22). Unlike the ProRS-fused INS domain, which relies on the anticodon-binding domain of ProRS to recognize tRNA^Pro^, the smaller free-standing *Cc* ProXp-ala domain recognizes the tRNA acceptor stem and aminoacyl moiety (22–24). ProXp-ala must distinguish misacylated Ala-tRNA^Pro^ from two cognate aa-tRNAs: Pro-tRNA^Pro^ and Ala-tRNA^Ala^. Three overlapping mechanisms have been proposed to contribute to the ability of ProXp-ala to discriminate between Ala- and Pro-tRNA^Pro^: conformational selection, size exclusion, and chemical selection (24).

Aminoacyl-tRNA synthetases recognize their cognate tRNAs by interacting with a specific set of nucleotides referred to as identity elements (25). The unique acceptor stem nucleotide G72 together with the discriminator base A73 are key elements that determine bacterial tRNA^Pro^ identity (26,27). In addition to their role in tRNA aminoacylation, previous studies showed that mutation of these tRNA^Pro^ acceptor stem elements also significantly decreased deacylation of Ala-tRNA^Pro^ by *Cc* ProXp-ala (22,23). The first base pair is more important for ProXp-ala discrimination of tRNA^Pro^ from tRNA^Ala^, since tRNA^Ala^ encodes a more common G1:C72 base pair but also possesses A73. The unique G3:U70 element in tRNA^Ala^ together with optimal binding by elongation factor-Tu, likely help ensure low levels of Ala-tRNA^Ala^ deacylation by ProXp-ala in the cell (22). No high-resolution structure of any *trans*-editing domain bound to its substrate aminoacyl-tRNA has been reported and specific interactions between elements on ProXp-ala and the tRNA^Pro^ acceptor stem have not been characterized.

In this work, we sought to further define the tRNA acceptor stem elements required for efficient deacylation by *Cc* ProXp-ala, and to identify the protein determinants of acceptor stem recognition. We compared two-dimensional ^1^H-^15^N-correlated NMR spectra of uniformly ^15^N-labeled (U-^15^N)-ProXp-ala in the presence of tRNA^Pro^ acceptor stem-derived RNAs containing the WT C1:G72 base pair or a terminal base pair transversion, G1:C72. We identified residues that likely interact with C1:G72, and performed deacylation and binding assays, which supported those conclusions. The role of the 3′ terminal tRNA A76 residue in positioning the aminoacyl moiety in the active site was also probed using binding and NMR studies. Taken together, these studies allow us to propose a detailed model for tRNA acceptor stem recognition by a *trans*-editing protein.

## MATERIALS AND METHODS

### Protein preparation

ProXp-ala mutagenesis was performed using the QuikChange Site-Directed Mutagenesis Kit (Agilent). All proteins were prepared as described previously (24). Briefly, the gene encoding *Cc* ProXp-ala and an N-terminal His_6_ tag was cloned into pET15b (Novagen) and proteins were expressed in *Ec* BL21-CodonPlus (DE3)-RIL cells (Agilent) upon induction with 0.1 mM isopropyl β-d-1-thiogalactopyranoside (Gold Biotechnology) for 20 h at room temperature. His_6_-tagged proteins were purified via His-Select Nickel Affinity Gel chromatography (Sigma-Aldrich) using a 5–250 mM imidazole elution. After buffer exchange into NMR buffer (50 mM sodium phosphate pH 7.5 and 150 mM NaCl), the His-tag was cleaved using a Thrombin Cleavage Capture Kit (Novagen) and the free His-tag as well as residual uncut His-tagged protein was removed using His-Select Nickel Affinity Gel chromatography. The resulting protein encodes full-length *Cc* ProXp-ala in addition to Gly-Ser-His N-terminal amino acids. For the final purification step, ProXp-ala was loaded onto a HiLoad 16/600 Superdex 75 size-exclusion chromatography (SEC) column (Cytiva). SEC was performed either in analytical ultracentrifugation (AUC) buffer (50 mM HEPES pH 6.8, 30 mM KCl, and 1 mM MgCl_2_) or NMR buffer. The Bio-Rad Protein Assay Kit was used to quantify protein concentrations. Uniform ^15^N labeling was achieved by culturing cells in M9 minimal media containing 1 g/l [^15^N]-ammonium chloride (Cambridge Isotopes) as the sole nitrogen source.

### RNA preparation

WT microhelix^Pro^, G1:C72-microhelix^Pro^, ΔA76-microhelix^Pro^, 3′pΔA76-microhelix^Pro^ and minihelix^Pro^ were purchased from Dharmacon (Sequences are found in Supplementary Table 1). RNAs were refolded at 10 μM in RNAse-free Milli-Q® water by heating at 80°C for 2 min then 60°C for 2 min, adding 10 mM MgCl_2_ and then placing on the bench to cool to room temperature. The refolded RNAs were buffer-exchanged into either AUC buffer via SEC, or NMR buffer via overnight dialysis. RNA concentration was quantified using Beer's Law by measuring absorbance at 260 nm (Nanodrop, Thermo Scientific) and using extinction coefficients ϵ_260_ provided by Dharmacon (28) (minihelix^Pro^, 330 300 M^−1^ cm^−1^; microhelix^Pro^, 201 400 M^−1^ cm^−1^; ΔA76-microhelix^Pro^, 187 600 M^−1^ cm^−1^).

Fluorescently-labeled duplex tRNA^Pro^ acceptor-TΨC stem analogs were prepared by annealing the fluorescently labeled 5′ fragments with equimolar concentrations of a complementary 3′ fragment. The sequences of these RNAs are shown in Supplementary Table 2. RNAs for these experiments were purchased from Sigma-Aldrich and the following extinction coefficients (28) were used: 5′ fragment, 140 700 M^−1^cm^−1^; 17-mer 3′ fragments, 161 400 M^−1^cm^−1^; 16-mer 3′ fragments, 147 600 M^−1^cm^−1^. The annealing was performed in NMR buffer as follows: RNA fragments were heated at 95°C for 5 min, slow cooled (1°C/min) to 45°C, annealed at 45°C for 30 min, and slow cooled (1°C/min) to 25°C.

The *Ec* tRNA^Pro^ and ΔA76-tRNA^Pro^ samples were prepared by *in vitro* transcription as previously described (24) with the exception that the DNA template for the ΔA76-tRNA^Pro^ transcription was PCR-amplified using Phusion polymerase (NEB) from a pUC119 plasmid encoding the gene for *Ec* tRNA^Pro^ and the following primers: 5′-TGCAGTAATACGACTCACTATAGGGAGAGCTACTCGCC-3′ (forward) and 5′-G[mG]TCGGCGAGAGAGGATTCGAACCTCC-3′ (reverse) (Sigma Aldrich). The second nucleotide of the reverse primer (bracketed) is 2′-*O*-methylguanosine, which attenuates the *N* + 1 activity of T7 RNA polymerase (29).

A non-hydrolyzable ACCA-3′-NH-Ala mimic containing an amide linkage was chemically synthesized as previously described (30).

To prepare 3′-[α-^32^P]-dA76-tRNA^Pro^, 15 μM ΔA76-tRNA^Pro^ (in a 100 μl reaction) was extended using 15 μM tRNA nucleotidyltransferase, 1 μM [α-^32^P]-dATP (Perkin Elmer) in 50 mM glycine pH 9.0, 10 mM MgCl_2_, 1 mM DTT and 1 mM pyrophosphate. Unlabeled dA76-tRNA^Pro^ was prepared using 5 μM dATP (Perkin Elmer) and the same reaction conditions as for the 3′-[α-^32^P]-substrate. WT 3′-[α-^32^P]- or unlabeled-tRNA^Pro^ (controls) were prepared with the same protocol using 1 μM [α-^32^P]-ATP or 5 μM ATP. The extended RNAs were purified by phenol-extraction and gel-filtration (G-25 quick spin column, Sigma-Aldrich), followed by ethanol precipitation. Unlabeled tRNA^Pro^ or 3′-dA76-tRNA^Pro^ were dissolved in water, quantified by measuring the absorbance at 260 nM and using an extinction coefficient of 604 000 M^−1^cm^−1^, adjusted to 100 μM, and combined with the precipitated [^32^P]-labeled tRNA^Pro^ or 3′-dA76-tRNA^Pro^, respectively. Aminoacylation reactions were performed at 4°C for 4 h using 25 μM tRNA, 25 μM dinitro-flexizyme (dFx) and 5 mM Ala-3,5-dinitrobenzyl ester (Ala-DBE) in 50 mM HEPES pH 7.5 and 600 mM MgCl_2_. The aminoacylated tRNAs were purified using a G-25 quick spin column, ethanol precipitated twice, dissolved in 3 mM NaOAc pH 5.2 and stored at -80°C. Ala-tRNA^Pro^, Ala-G1:C72-tRNA^Pro^ and Ala-A73C-tRNA^Pro^ were prepared as previously described (23).

### Deacylation assays

The Ala-dA76-tRNA^Pro^ and Ala-A76-tRNA^Pro^ (control) deacylation reactions were performed at room temperature in 50 mM HEPES pH 6.8, 20 mM KCl, 5 mM MgCl_2_, 0.1 mg/ml BSA, while reactions of Ala-G1:C72-tRNA^Pro^ and Ala-A73C-tRNA^Pro^ were assayed together with WT Ala-tRNA^Pro^ (control) at 18°C in 150 mM potassium phosphate pH 7.0, 5 mM MgCl_2_, and 0.1 mg/mL BSA. All reactions were performed in triplicate under single-turnover conditions (0.1 μM substrate tRNAs and 0.75 μM enzyme). At the indicated time points (0–30 min), 2 μl reaction aliquots were quenched by mixing with equal volume of quenching buffer (200 mM sodium acetate, pH 5.2, containing 20 units of S1 nuclease). The S1-digested products, Ala-AMP (or Ala-dAMP), were separated from AMP (or dAMP) by thin layer chromatography (TLC) using polyethyleneimine-cellulose plates (EMD Millipore) and 0.05% ammonium chloride/5% acetic acid as a mobile phase. The plates were imaged using a Typhoon FLA 9500 phosphorimager (Cytiva) and amount of substrate and product was quantified via ImageQuant TL 8 software (Cytiva). After correcting for nonenzymatic buffer hydrolysis at each timepoint, deacylation data were analyzed using GraphPad Prism and *k_obs_* values were obtained by fitting time courses of the substrate disappearance to either one- or two-phase exponential decay equations. Double-exponential fits were only required to fit three sets of assays: WT ProXp-ala and WT or A73C tRNA^Pro^, and K50A ProXp-ala and WT tRNA^Pro^. The *k_obs_* values from the fast phases, which represented at least 80% of the amplitude, were used for all the comparisons (see Supplementary Table 3). Reported *k_obs_* values are averages from three independent experiments.

### Analytical ultracentrifugation (AUC)

AUC was performed as previously described (24). Briefly, absorbance at 260 nM was monitored in sedimentation velocity experiments conducted at 50000 rpm (182 000 × g at cell center and 201 600 × g at cell bottom) at 20°C in AUC buffer using an An50 Ti rotor and standard double-sector Epon centerpieces equipped with sapphire windows. Samples for AUC experiments contained 1 μM microhelix^Pro^ and various concentrations of ProXp-ala (1.2, 2.4, 4.8 and 9.6 μM); 70 scans were collected over a 10-h period for each sample. The AUC data were fit to a continuous *c*(*s*) distribution using SEDFIT v. 15.01 (sedfitsedphat.nibib.nih.gov) (31–33). The partial specific volume of the RNA was taken to be 0.55 cm^3^/g (34). The sedimentation coefficient distributions, *c*(s), were integrated to obtain the signal-weighted average sedimentation coefficients (*s*_w_). Relative binding affinities of ProXp-ala mutants were estimated from comparison to the *s*_w_ values of apo microhelix^Pro^ (*s*_w_ = 1.76) and microhelix^Pro^ in the presence of two concentrations of WT ProXp-ala: 1.2 μM (*s*_w_ = 2.24) and 9.6 μM (*s*_w_ = 2.71). The ProXp-ala concentrations of 0, 1.2 and 9.6 μM correspond to 0, 36%, 85% RNA bound states (calculated from binding to a quadratic equation using the previously reported *K*_D_ value (24)).

### Nuclear magnetic resonance (NMR) spectroscopy studies

Protein and RNA samples used in NMR experiments were prepared as described above. [U-^15^N]-ProXp-ala (100 μM) was mixed with equimolar RNA (WT microhelix^Pro^, G1:C72-microhelix^Pro^, ΔA76-microhelix^Pro^, 3′pΔA76-microhelix^Pro^ and minihelix^Pro^) separately in NMR buffer containing 10% D_2_O (vol/vol) as the lock signal and 0.001% 4,4-dimethyl-4-silapentane-1-sulfonic acid (DSS, wt/vol) as the reference signal. NMR samples (550 μl) were transferred to a 5 mm NMR tube (Wilmad). The HSQC spectra were recorded at 25°C on a Bruker DRX-800 spectrometer equipped with a TXI cryoprobe, and processed and analyzed using NMRFx and NMRViewJ (35,36). Backbone amide assignments for ProXp-ala were inferred by comparison to the assignments of the free and microhelix-bound protein (24). The ^1^H chemical shifts were directly referenced with DSS while ^15^N chemical shifts were indirectly referenced. Combined chemical shift perturbations (CSPs) were calculated from weighted differences in ^1^H and ^15^N chemical shifts: Δδ (ppm) = (Δδ_H_^2^ + Δδ_N_^2^/25)^1/2^.

### Electrophoretic mobility shift assays (EMSAs)

For EMSAs, 81 nM annealed RNA duplex was incubated for 1 h at room temperature with different amounts of ProXp-ala (0–114 μM), and then mixed with 6x native gel loading dye (NEB). The samples were run on a 16% native polyacrylamide gel at 120 V for 1 h in Tris/borate/EDTA (TBE) buffer at room temperature. The gels were imaged using a Typhoon FLA 9500 phosphorimager and fluorescence intensities of RNA bands were quantified using ImageQuant TL 8 software. Because of smearing of the bands corresponding to the RNA/ProXp-ala complex, bound fractions were inferred from depletion of bands corresponding to the free RNA [1 – (free/total RNA)]. Apparent equilibrium dissociation constants, *K*_D_, were obtained from fits to the Hill equation using Microsoft Excel Solver. Each *K*_D_ value was based on the average of three independent replicates.

### Model building

A model of an aminoacylated truncated microhelix^Pro^ charged with alanine was prepared starting from a previous docking model, which contains a 5′-CCA-Ala ligand as the starting point (24). The two 5′ terminal cytidines were removed. For the truncated microhelix^Pro^, a 3D model with sequence 5′-CGGUUCGCCGACCA-3′ was prepared using RNAComposer (37). The truncated microhelix^Pro^, which has a 3-base pair stem and 4-nt tetraloop, was manually adjusted using Chimera such that the 5′ phosphate of the 3′ terminal adenosine aligned with the 5′ phosphate of the adenosine ligand from the previous docking model (24) and the G10 residue of the microhelix (equal to G72 in full-length tRNA^Pro^) was close to R80 of ProXp-ala (38). The subsequent model was then loaded into Coot and the R80 geometry was adjusted using the rotamer tool such that it had the correct geometry to hydrogen bond with the Hoogsteen edge of G10 (39). The resulting structure was minimized and subjected to a short 10-ns explicit solvent molecular dynamics simulation using AmberTools 21 and AMBER 20 to allow the RNA-protein complex to adjust to the bound state (40).

All simulation steps (minimization, equilibration, and production) were carried out using the AMBER ff14SB force field (41). To prepare the model for simulation, it was first neutralized with sodium ions and solvated with an octahedral TIP3P water box with a 10 Å cutoff (42). Minimization was conducted in two steps. First, 5000 steps were performed with 1000 steps of steepest descent with constant volume periodic boundaries and 500 kcal/mol·Å^2^ positional restraint on the solute atoms (43). Second, 5000 steps were performed with 1000 steps of steepest descent with the entire system unrestrained. In the first equilibration step, the system was gradually heated from 0 K to 300 K over 200 ps with the solute restrained with a weak, 10 kcal/mol·Å^2^, positional restraint. The same parameters were used for the 800 ps second equilibration step and the 10 ns production step; only the residues representing the ligand-binding sites (residues defining the alanine binding site as well as R80 and G10) were restrained with weak, 10 kcal/mol/Å^2^, positional restraints. These steps were performed using constant pressure periodic boundary conditions, the SHAKE algorithm to constrain all bonds involving hydrogen, and Langevin dynamics applied at a collision frequency of 1.0 ps^−1^ to maintain temperature (44). A random seed was used for all equilibration and production simulations to eliminate potential synchronization artifacts of the simulations that may result from the use of Langevin dynamics (45,46). A nonbonded cutoff of 8.0 Å was used for all steps.

## RESULTS

### ProXp-ala recognizes tRNA^Pro^ acceptor stem elements via Arg80

**Figure 1.**
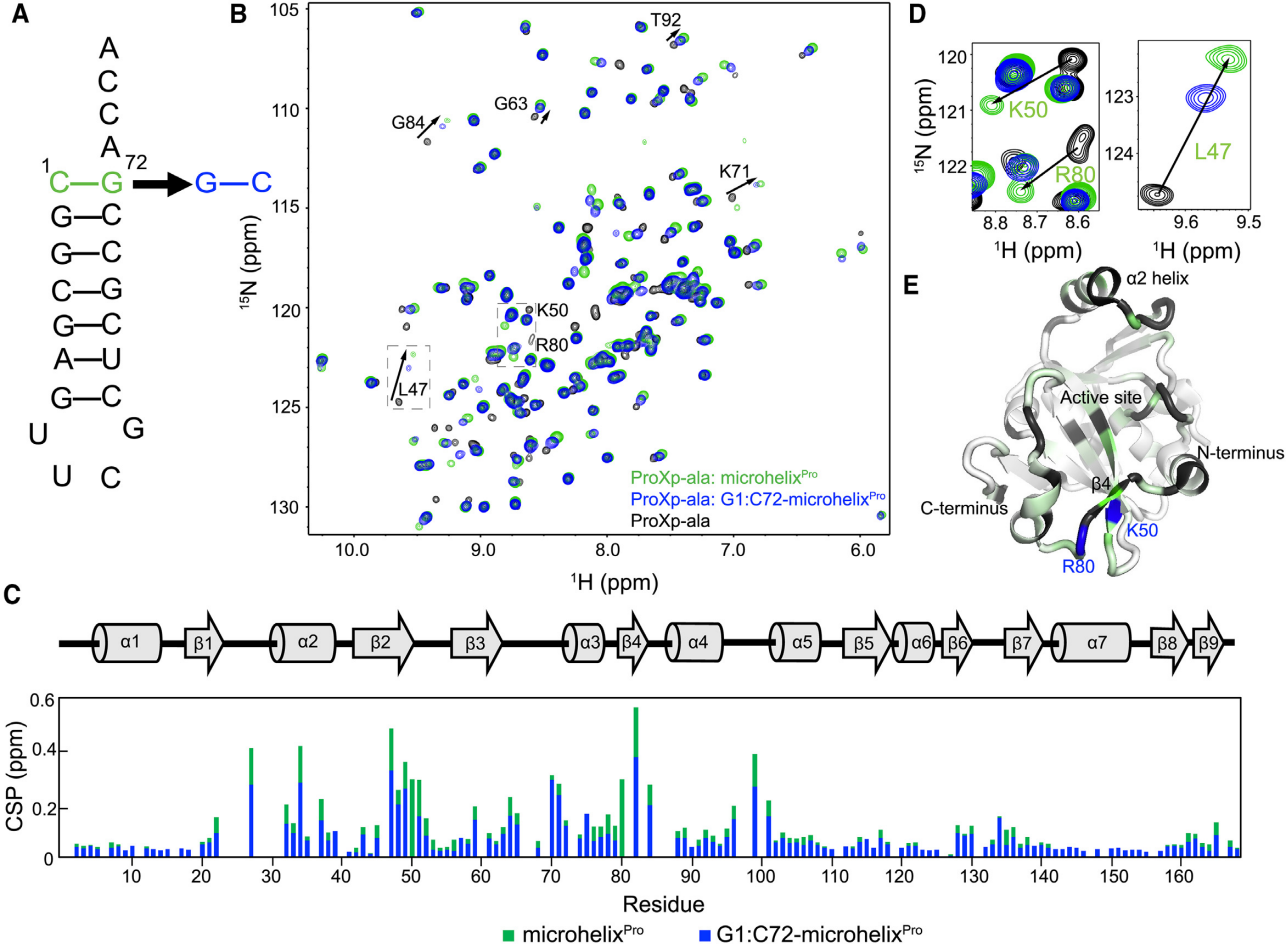
NMR Chemical shift perturbations (CSPs) implicate Lys50 and Arg80 of *Cc* ProXp-ala in tRNA^Pro^ recognition. (**A**) Secondary structure of microhelix^Pro^ with the WT C1:G72 base pair (green) and the G1:C72 variant (blue). (**B**) Overlaid ^1^H-^15^N HSQC spectra of [U-^15^N]-ProXp-ala (black) alone and in the presence of WT- (green) or G1:C72-microhelix^Pro^ (blue). Perturbations of select residues are labeled and indicated by arrows. (**C**) Secondary structure of *Cc* ProXp-ala (top) and magnitude of per residue CSPs induced by WT- (green) and G1:C72-microhelix^Pro^ (blue) (bottom). (**D**) Spectral expansions highlighting CSPs for Lys50 and Arg80 (left) and Leu47 (right). (**E**) CSP differences between WT- and G1:C72-microhelix^Pro^-bound states mapped with a linear color gradient from white (0 ppm) to dark green (0.25 ppm) on a cartoon representation of the *Cc* ProXp-ala crystal structure (PDB: 5VXB). Unassigned residues are grey, and Lys50 and Arg80 are highlighted in blue.

To identify *Cc* ProXp-ala residues responsible for recognizing Ala-tRNA^Pro^, we collected NMR spectra of [U-^15^N]-ProXp-ala alone and in the presence of tRNA^Pro^ analogues. The microhelix^Pro^ analogue and its derivatives include the tRNA^Pro^ acceptor stem and a UUCG tetraloop (Figure 1A). Uncharged WT microhelix^Pro^ binds ProXp-ala with only a 3-fold lower affinity compared to the charged Ala-microhelix^Pro^ substrate and retains all other features important for recognition by ProXp-ala (24). To identify residues responsible for recognition of the terminal base pair (C1:G72), we also investigated G1:C72-microhelix^Pro^ bearing a base pair transversion (Figure 1A). The 2D ^1^H–^15^N HSQC spectra of ProXp-ala alone and in a complex with WT or G1:C72-microhelix^Pro^ are shown in Figure 1B. Comparison of the chemical-shift perturbations (CSPs) induced by WT microhelix^Pro^ and the G1:C72 variant showed that CSPs were generally observed for the same signals and in the same direction (Δδ_H_/Δδ_N_), but of lower magnitude for the mutant (Figure 1B, C). This pattern is consistent with isosteric but weaker binding of G1:C72-microhelix^Pro^, as illustrated by the CSPs induced on Leu47 (Figure 1D, right). However, no resonances corresponding to Arg80 and Lys50 could be discerned in the G1:C72-microhelix^Pro^-bound spectrum. We infer that the chemical environments for those resonances are quite different when bound to C1:G72- or G1:C72-microhelix^Pro^ (Figure 1D, left). Together with their proximal location near the entrance to the active site cavity (Figure 1E), these findings are consistent with roles for Arg80 and Lys50 in recognition of the C1:G72 base pair.

**Figure 2.**
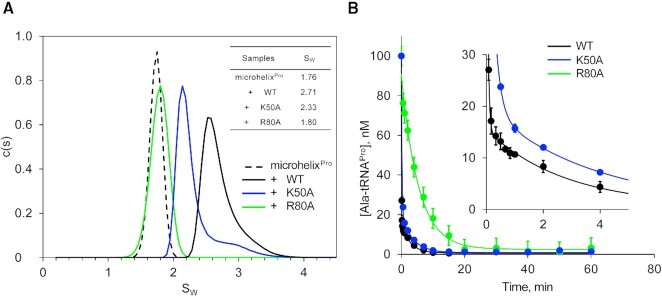
Arg80 and Lys50 are critical for both RNA binding and substrate deacylation. (**A**) Analytical ultracentrifugation sedimentation velocity analysis of 1 μM WT microhelix^Pro^ alone (dashed line), and in the presence of 9.6 μM WT (black), R80A (green) or K50A (blue) ProXp-ala. Inset table shows s_w_ values obtained from integrated *c*(*s*) distributions. (**B**) Single-turnover deacylation assays of 0.1 μM Ala-tRNA^Pro^ by 0.75 μM WT (black), R80A (green) and K50A (blue) ProXp-ala. Inset shows an expanded view of the WT and K50A ProXp-ala data. Lines represent single- (R80A) and double- (K50A and WT) exponential fits of the data. Error bars are the standard deviation of three replicates. All deacylation analyses were performed after correcting for nonenzymatic buffer hydrolysis at each timepoint.

Informed by the unique NMR spectral pattern for Lys50 and Arg80, we individually tested the effect of Ala mutation of these residues in RNA binding and catalytic function of *Cc* ProXp-ala. We applied AUC to determine s_w_ of microhelix^Pro^ alone and in the presence of WT ProXp-ala and the K50A- or R80A variants (Figure 2A). Relative binding affinity was estimated from comparison of the signal-weighted average s_w_ obtained from integrated c(s) distributions. Sedimentation velocity data for 1 μM microhelix^Pro^, monitored at 260 nm where the signal is dominated by the RNA, yielded a *s*_w_ of 1.76. Sedimentation velocity experiments in the presence of 1.2 and 9.6 μM WT ProXp-ala proteins resulted in *s*_w_ values of 2.24 and 2.71. Based on the previously determined K_D_ value of 1.5 μM for binding of WT ProXp-ala to microhelix^Pro^ (24), this would suggest 36% and 85% bound states for the concentrations of *Cc* ProxP-ala used in AUC experiments. Since 9.6 μM K50A is required to reach a *s*_w_ value close to 36% bound, we estimate that the binding affinity of K50A is approximately 8-fold weaker than WT ProXp-ala. The effect of the R80A substitution is even more severe; the presence of 9.6 μM R80A resulted in a *s*_w_ value similar to that of apo microhelix^Pro^, indicating a very weak interaction with RNA. Single-turnover deacylation assays showed that K50A hydrolyzed Ala-tRNA^Pro^ with a 4-fold reduced rate compared to WT protein, whereas R80A showed ∼117-fold decreased deacylation activity (Figure 2B). These data confirmed important roles for Arg80 and Lys50 in tRNA^Pro^ recognition.

**Figure 3.**
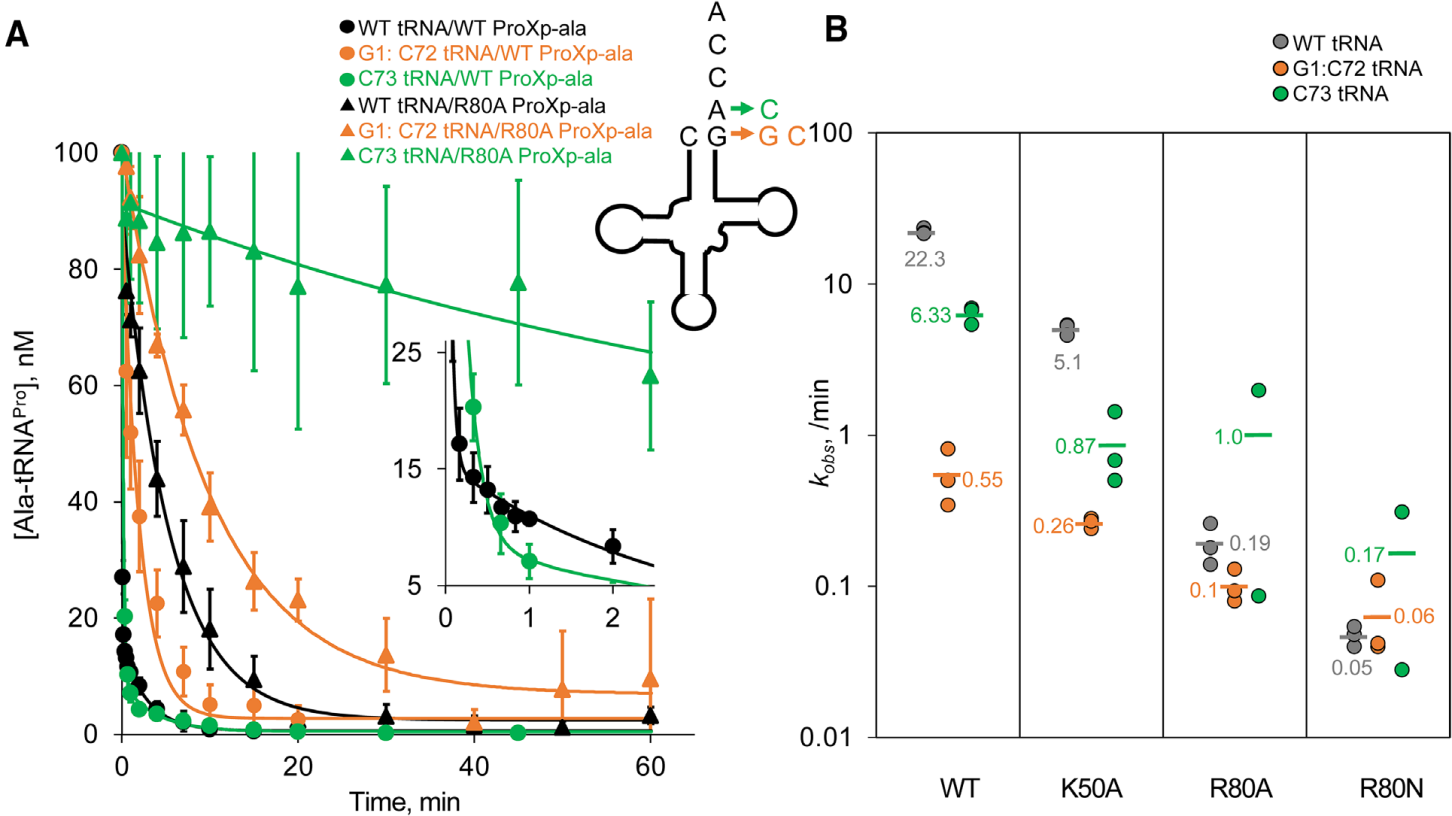
Single-turnover deacylation assays show that K50A and R80A ProXp-ala variants display reduced sensitivity to tRNA^Pro^ acceptor stem mutants compared to WT ProXp-ala. (**A**) Single-turnover deacylation assays performed with WT (closed circles) or R80A ProXp-ala (open circles), using either WT (black), G1:C72- (orange), or C73- (green) Ala-tRNA^Pro^. Lines represent fits to a double-exponential equation in the case of WT ProXp-ala and WT or A73C tRNA^Pro^; the other lines represent single-exponential fits of the data. Error bars are the standard deviation of three replicates. All deacylation analyses were performed after correcting for nonenzymatic buffer hydrolysis at each timepoint. (**B**) Summary of *k_obs_* values (min^−1^) for WT, K50A, R80A and R80N ProXp-ala, and WT (gray), G1:C72-(orange) and C73-(green) tRNA^Pro^. In cases where double-exponential fits were used (WT ProXp-ala and WT or A73C tRNA^Pro^; K50A ProXp-ala and WT tRNA^Pro^), *k_obs_* values were obtained from the fast phase, which represented at least 80% of the amplitude (see Supplementary Table 3).

To evaluate whether Arg80 and Lys50 specifically recognize the C1:G72 base pair, we compared the deacylation rates of WT and G1:C72-substituted Ala-tRNA^Pro^ catalyzed by WT, K50A and R80A ProXp-ala under single turnover conditions (Figure 3). For WT ProXp-ala, G1:C72 substitution results in a 41-fold lower deacylation rate compared to WT Ala-tRNA^Pro^. The K50A mutation had decreased deacylation rates for all three Ala-tRNA^Pro^ substrates investigated here and was 2-fold less sensitive to G1:C72 substitution relative to WT ProXp-ala; this base pair transversion resulted in a 20-fold decrease in deacylation rate relative to WT tRNA^Pro^. The R80A mutation strongly diminished enzyme activity, reflecting the role of Arg80 in promoting tRNA^Pro^ binding (Figure 2). Neither Lys50 nor Arg80 is strictly conserved in the INS superfamily. Human ProXp-ala encodes Lys50 but has an asparagine at the location of Arg80; thus, we also tested the activity of an R80N mutant. The results showed that the R80A/N variants were unable to effectively discriminate against the G1:C72 variant, catalyzing deacylation of WT and G1:C72 substrates with similar rates (Figure 3).

Previous data obtained under multiple turnover conditions showed that A73 also plays an important role in tRNA^Pro^ deacylation by *Cc* ProXp-ala (23). WT ProXp-ala showed a 3.5-fold decrease in deacylation upon A73C mutation under the conditions used here, whereas K50A ProXp-ala was even more sensitive (∼ 6-fold decrease) to this change (Figure 3). Interestingly, the A73C mutation had a positive impact on both R80A and R80N substitutions with 3- to 5-fold increased deacylation rates compared with WT tRNA^Pro^. Taken together, these data suggest that Arg80 is likely a direct interaction partner of C1:G72, whereas Lys50 may play a less direct role in first base pair or A73 recognition.

**Figure 4.**
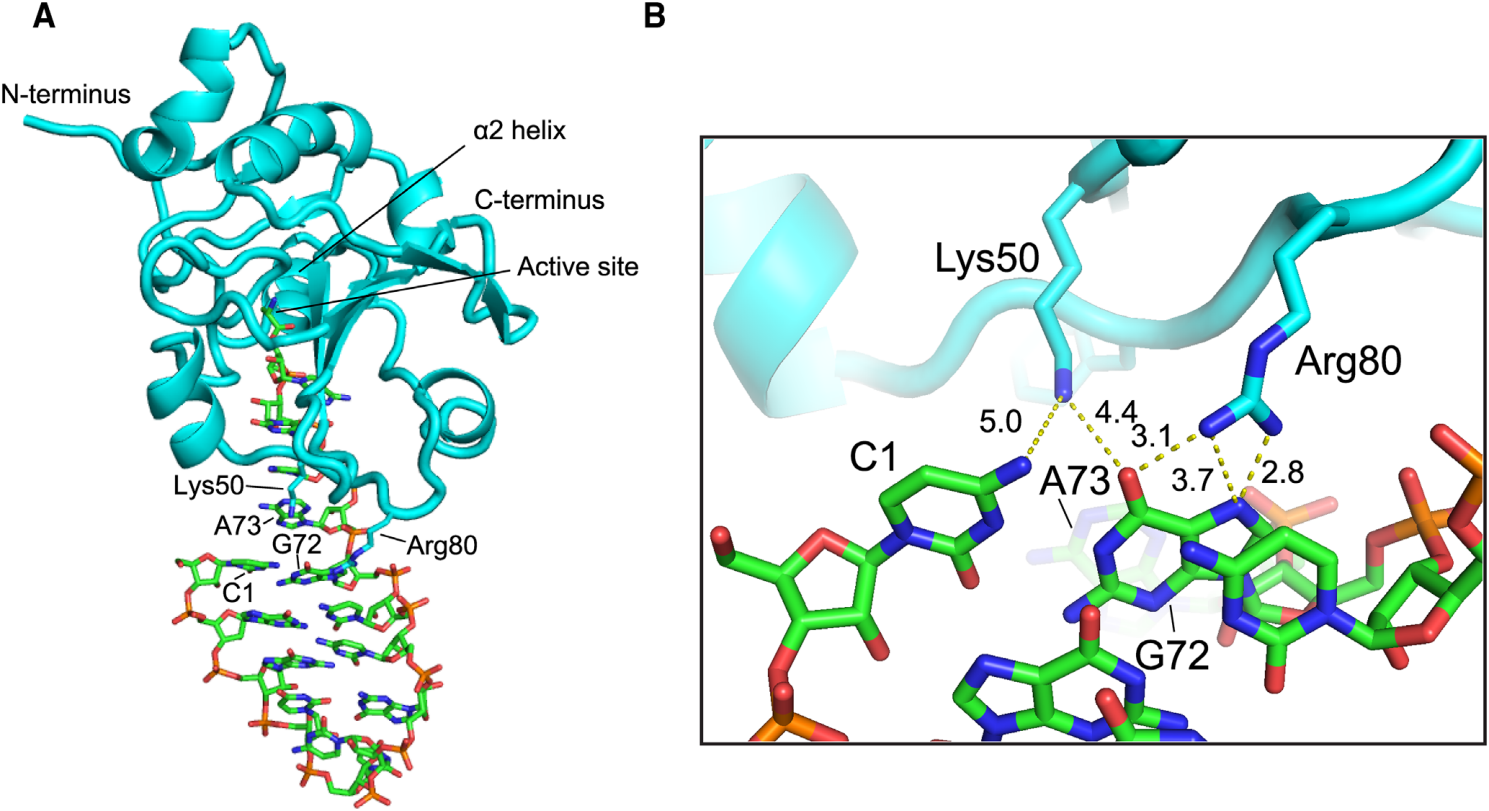
Docking model of the truncated microhelix^Pro^-ProXp-ala complex shows inferred interactions between Arg80 and Lys50 side chains and the C1:G72 base pair major groove. Overview (**A**) and zoomed in view (**B**). Distances in Å between Lys50 Nζ and Arg80 Nη atoms and C1 N4 and G72 N7 and O6 are indicated by dashed lines.

Guided by NMR, binding and deacylation results, we built an atomic model of ProXp-ala bound to a truncated microhelix^Pro^ containing the ACCA-Ala single-stranded region, the top 3 base pairs of the acceptor stem, and a closing tetraloop (Figure 4). As a starting point, we used an existing docking model of ProXp-ala in complex with 5′-CCA-Ala (24). We constrained G72 to be proximal to Arg80 and then performed a 10 ns molecular dynamics simulation to allow relaxation of the bound state. In this model ProXp-ala interacts with the tRNA acceptor end, with the O6 and N7 of G72 positioned for hydrogen bonding to the Arg80 side chain. This model therefore accommodates direct interactions between Arg80 and the major groove of C1:G72 without significant RNA or protein distortion. Lys50 is slightly further removed from the first base pair (∼5 Å) and does not appear to directly interact with A73, according to this model and in good agreement with the deacylation data.

### Essential interaction between lys45 of ProXp-ala and A76 in tRNA^Pro^

While C1:G72 is crucial for selection of tRNA^Pro^ versus tRNA^Ala^, as the attachment point for aminoacylation, positioning of A76 into the active site is critical for tRNA binding and deacylation. Lys45 of *Cc* ProXp-ala is strictly conserved throughout the INS superfamily (17,18,24,47,48) and mutation of this Lys to Ala in all family members tested to date dramatically impairs deacylation activity (17,18,24). Computational studies support a model in which this residue directs the aminoacylated A76 into the active site via interactions with the phosphate group between A76 and C75 (47–49). To study the interaction of Lys45 with the tRNA^Pro^ acceptor stem using experimental structural approaches, we prepared a microhelix^Pro^ construct lacking the terminal A76 residue (ΔA76-microhelix^Pro^, Supplementary Figure S1A) and performed NMR CSP analysis (Supplementary Figure S1B and S1C). In contrast to the WT or G1:C72-microhelix^Pro^, only minor CSPs were induced in the spectrum of ProXp-ala in the presence of 100 μM ΔA76-microhelix^Pro^ (Supplementary Figure S1C), indicating minimal binding of this variant and confirming that A76 is an essential tRNA binding determinant.

**Figure 5.**
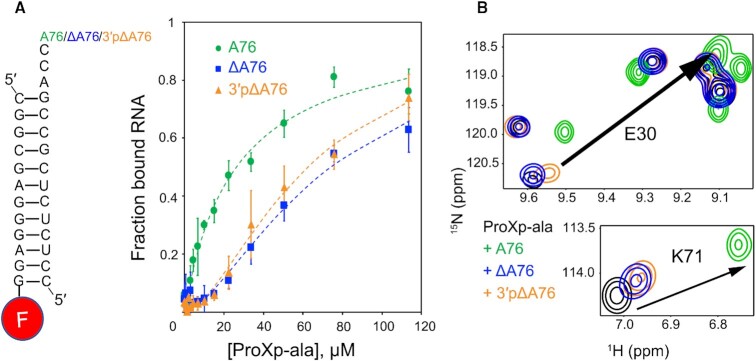
ProXp-ala binding to tRNA^Pro^ is sensitive to changes in A76. (**A**) Left: schematic of RNA duplex variants used in EMSA assays where one strand is fluorescently labeled at the 3′ end. Right: Results of EMSA binding assays plotting fraction of bound RNA as a function of ProXp-ala concentration; A76 (WT, green), ΔA76 (blue) and 3′pΔA76 (orange). Lines are Hill equation fits to the data; error bars are standard deviation of three replicates. (**B**) Overlaid ^1^H-^15^N HSQC spectra for the amide nitrogens of Glu30 (top) and Lys71 (bottom) illustrate decreased CSPs upon A76 mutations: Free [U-^15^N]-ProXp-ala (black), [U-^15^N]-ProXp-ala in the presence of the ΔA76-microhelix^Pro^ (blue), 3′pΔA76-microhelix^Pro^ (orange), or WT (A76) microhelix^Pro^ (green). The arrows indicate the CSPs observed upon WT microhelix^Pro^ binding to free ProXp-ala.

To further investigate the contribution of A76 to tRNA binding by ProXp-ala, we performed EMSA binding experiments using RNA duplexes corresponding to the tRNA^Pro^ acceptor-TΨC stems. To form the duplexes, a 3′ fluorescently-labeled 5′ fragment was annealed to 3′ fragments with varying 3′ ends: A76, ΔA76, 3′pΔA76, A76C, A76C, A76U or dA76 (Figure 5A, Supplementary Figure S2 and Supplementary Table 2). This strategy was chosen to avoid a bulky probe near the 3’ acceptor end, which is critical for binding. The affinity of ProXp-ala for WT microhelix^Pro^ was previously reported to be 0.97 and 1.5 μM from NMR and AUC experiments (24), respectively. In the EMSA experiments, the best fit for fractional binding to a Hill binding equation yielded an apparent *K*_D_ of 33 μM. These differences in *K*_D_ values likely result from the use of different RNA substrates (duplexes versus hairpin microhelices) and the fact that EMSA is a nonequilibrium technique, while in NMR and AUC studies, complexes most likely exist in fast equilibrium (24). Nevertheless, relative differences in binding observed in EMSAs are still informative.

As summarized in Table 1, with the exception of the 2′-deoxy-A76 variant (dA76), the other six A76 duplex RNA variants exhibited weaker binding to ProXp-ala than WT duplex^Pro^. Deletion of A76 resulted in a 2-fold decrease in binding (Figure 5A and Table 1). Comparison of ProXp-ala binding to ΔA76 and 3′pΔA76 suggests that the 3′ phosphate does not contribute significantly to the binding affinity in the absence of A76 (Figure 5A and Table 1). Based on NMR CSP experiments, spectra recorded in the presence of 3′pΔA76-microhelix^Pro^ also exhibited very similar CSPs relative to spectra in the presence of ΔA76-microhelix^Pro^ (Figure 5B). A76C and A76U microhelix^Pro^ variants showed the lowest affinity for ProXp-ala, while A76G variant binding was slightly higher, similar to that of ΔA76 and 3′pΔA76 (Table 1). These data indicate that position 76 purine ring properties are important for ProXp-ala binding.

**Table 1.** Apparent *K*_D_ values for ProXp-ala binding to A76 duplex^Pro^ variants as determined from EMSAs. Values were determined from at least three independent trials as explained in the Materials and Methods

Variant	Apparent *K*_D_ (μM)
A76 (WT)	33 ± 11
dA76	34 ± 5
△A76	75 ± 12
3′p△A76	63 ± 11
A76C	87 ± 4
A76G	66 ± 11
A76U	106 ± 16

**Figure 6.**
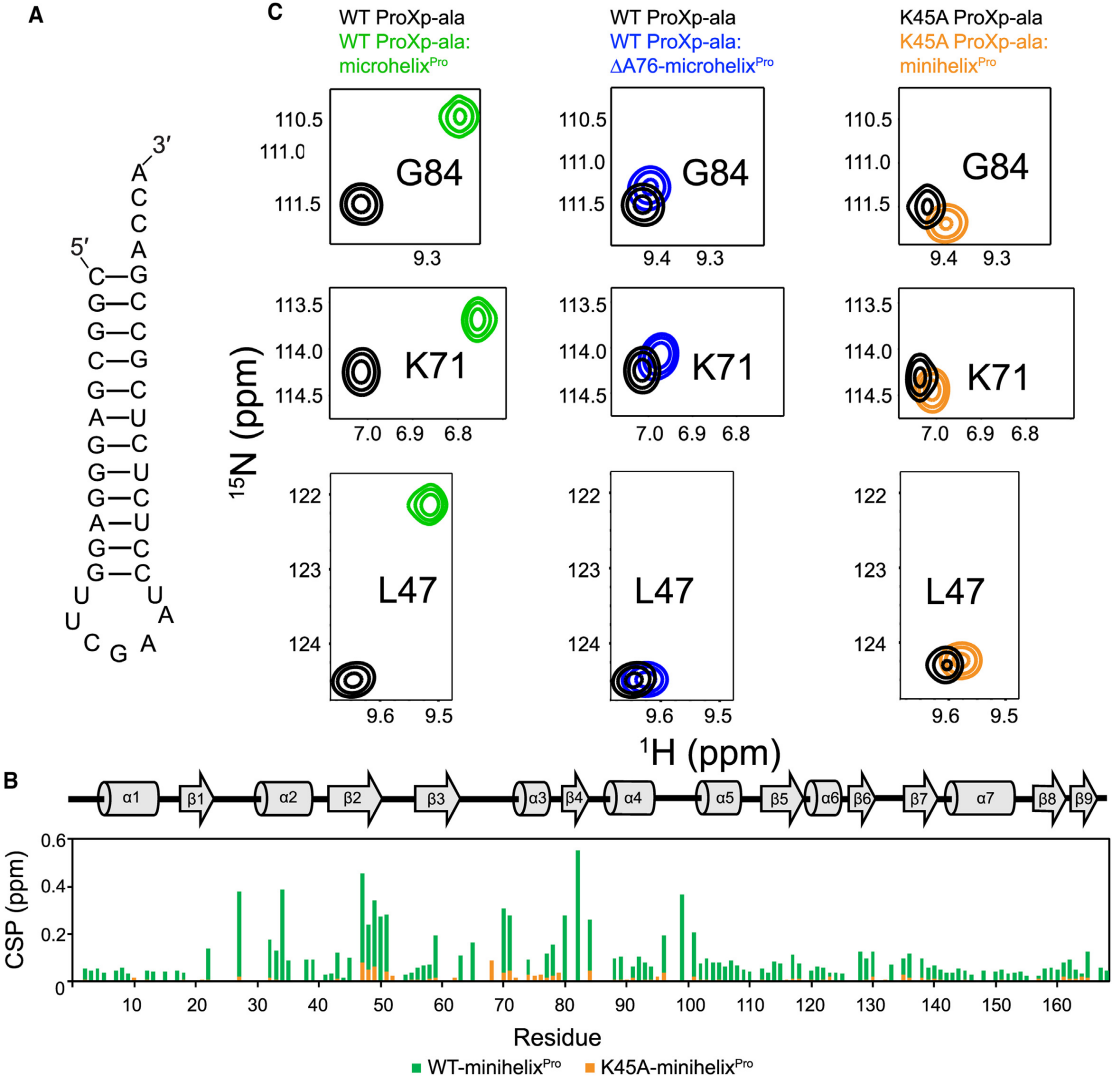
CSPs show parallel ProXp-ala-tRNA^Pro^ binding deficiencies upon deletion of A76 and mutation of K45. (**A**) Schematic of minihelix^Pro^, which is indistinguishable from microhelix^Pro^ in terms of its binding to ProXp-ala (Supplementary Fig. 4). (**B**) Secondary structure of *Cc* ProXp-ala (top) and summary of per residue CSPs induced by minihelix^Pro^ binding to WT (green) and K45A (orange) ProXp-ala. (**C**) Spectral expansions of Gly84 (top), Lys71 (middle) and Leu47 (bottom) from overlaid ^1^H–^15^N HSQC spectra of WT ProXp-ala (left and middle spectra) free (black), and in the presence of microhelix^Pro^ (green) or ΔA76-microhelix^Pro^ (blue), and of K45A ProXp-ala (right spectra) free (black), and in the presence of minihelix^Pro^-bound (orange).

We tested the role of Lys45 in A76 recognition by alanine mutagenesis. Previous AUC studies performed at low micromolar concentration failed to detect binding by K45A ProXp-ala to microhelix^Pro^ (24). Here, we used NMR to determine whether this mutation induces any structural defect, and to test binding to a larger minihelix^Pro^ at higher concentrations. This longer construct, minihelix^Pro^, is indistinguishable from microhelix^Pro^ in terms of its binding to ProXp-ala (Supplementary Figure S4). The 2D ^1^H–^15^N spectrum of K45A ProXp-ala revealed no folding defect, with CSPs between WT and the K45A variant limited to the vicinity of Lys45, including a significant shift of the G63 peak (Supplementary Figure S3). The NMR spectrum of K45A ProXp-ala in the absence and presence of WT minihelix^Pro^ (Figure 6A) revealed small CSPs upon RNA addition that reflect a similar manner of RNA recognition, but overall reduced binding (Figure 6B). The combined findings of diminished binding to the WT partner upon either K45A mutation of ProXp-ala or A76 truncation of microhelix^Pro^ are consistent with K45-A76 interaction.

### The 2′OH of A76 contributes to ProXp-ala catalysis

**Figure 7.**
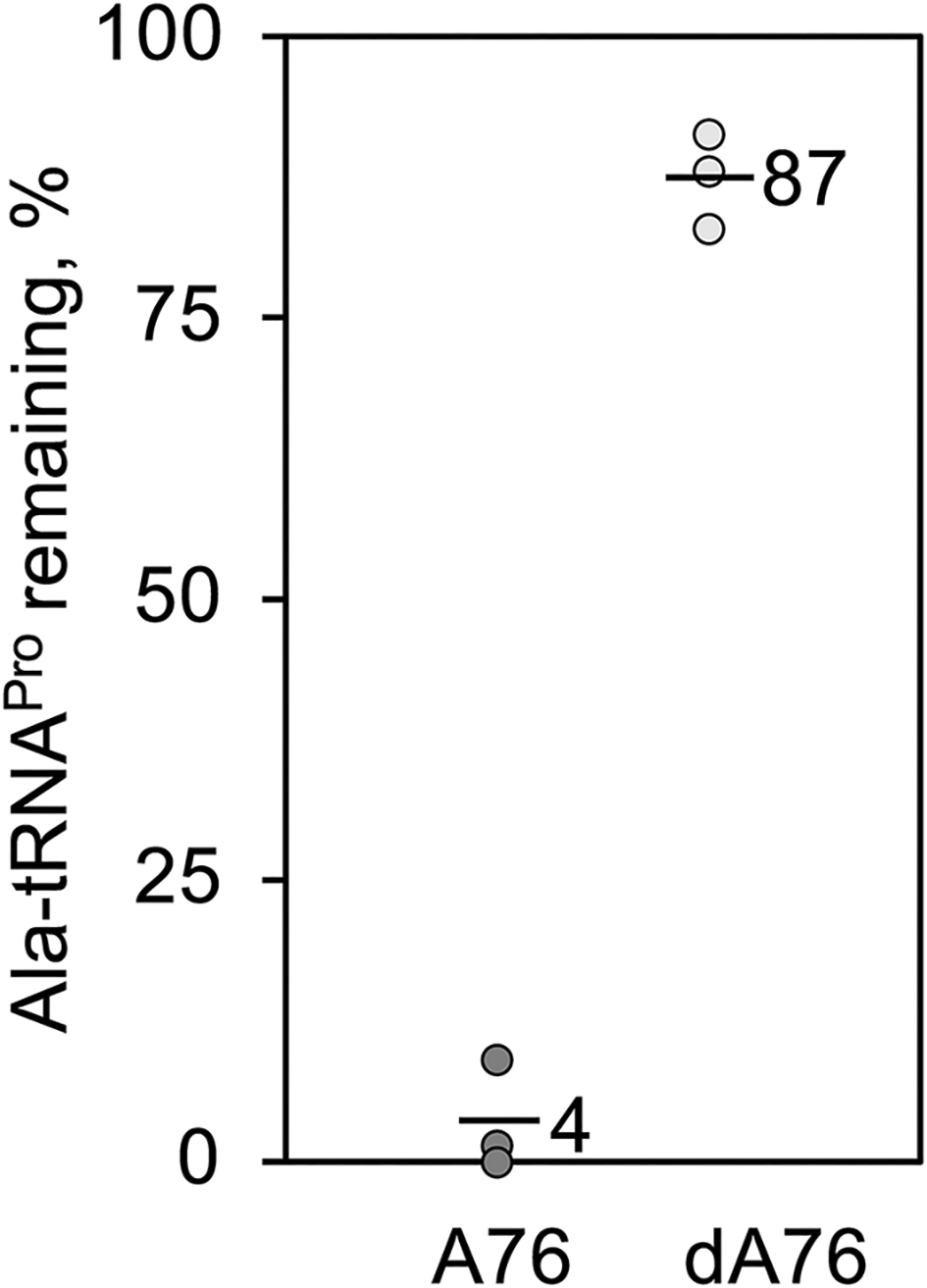
The 2′ OH of A76 is important for deacylation of Ala-tRNA^Pro^ by ProXp-ala. Deacylation assay with 0.75 μM ProXp-ala and 0.1 μM Ala-A76-tRNA^Pro^ (dark grey) or Ala-dA76-tRNA^Pro^ (light grey). Assays were performed in triplicate with the mean value of aminoacyl-tRNA remaining after 30 min indicated by the top of the bar.

Previous computational studies on the INS domain have suggested that bacterial ProRS deacylates Ala-tRNA^Pro^ using a water-mediated mechanism involving a backbone carbonyl and the 2′OH of A76 (50). A similar mechanism has been proposed for *Cc* ProXp-ala but not directly tested (24). Deacylation assays carried out using *Cc* ProXp-ala and a tRNA substrate lacking the A76 2′OH, Ala-dA76-tRNA^Pro^, indeed implicate this functional group in catalysis. Compared to the WT substrate, which is 96% deacylated after 30 min, only 13% deacylation of Ala-dA76-tRNA^Pro^ is observed at the same time point (Figure 7). Considering the EMSA results, which showed that dA76-duplex^Pro^ binds to ProXp-ala with the same affinity as the WT duplex (Table 1), we conclude that the functional role of the A76 2′OH is primarily to facilitate catalysis, not substrate binding.

## DISCUSSION

Using NMR spectroscopy to examine the interactions between *Cc* ProXp-ala and the acceptor stem of WT and mutant tRNA^Pro^, we identified two residues, Arg80 and Lys50, as likely candidates for binding to the unique C1:G72 pair and/or A73 nucleotide, which were previously identified as key recognition elements. We showed that Arg80 was critical for tRNA binding and deacylation and results suggested a direct interaction between this residue and the first base pair. Computational modeling results supported a direct Arg80–G72 major groove hydrogen bonding interaction. The K50A ProXp-ala variant remained sensitive to changes at both A73 and C1:G72, and the modeling studies do not support a direct interaction with the discriminator base; our model predicts a close interaction (∼5 Å) between K50 and functional groups in the first base pair. Consistent with the importance of C1:G72 for substrate binding, ACCA^76^-nh-Ala, a small nonhydrolyzable amide-linked 3′-end tRNA mimic containing both A76 and the discriminator base A73 but lacking the top of the acceptor stem, failed to inhibit the deacylation of Ala-tRNA^Pro^ by ProXp-ala (data not shown).

Arg80 resides on the loop preceding the β4 strand, adjacent to the active site of ProXp-ala (Figure 1E). In previous NMR studies, the signal for Arg80 appeared at a similar location in microhelix^Pro^- and Ala-microhelix^Pro^-bound ProXp-ala spectra, which implied that Arg80 mainly interacted with the RNA and not the alanyl moiety (24), a conclusion supported by the new results. Arg80 is also one of the residues that exhibited chemical exchange in the μs-ms regime in previous studies, suggesting it may be involved in the conformational sampling that enables substrate binding and selectivity (24).

Interaction of the Arg guanidinium group with the major groove of guanine is a common mechanism for site-specific RNA-protein interaction (51–55). The G72 nucleotide of bacterial tRNA^Pro^ is a critical recognition element for both ProRS and the ProXp-ala *trans*-editing domain. Based on kinetic data and a novel cross-linking approach, we previously identified a specific hydrogen bonding interaction between an Arg side chain in the active site of *E. coli* ProRS (R144 in the conserved motif 2 loop) and the major groove functional groups of G72 (56). Changes at R144 did not substantially alter the Michaelis constant for tRNA, but significantly affected the *k*_cat_ parameter. Since ProRS also strongly recognizes the tRNA anticodon, we proposed that the R144–G72 contact plays a critical role in an ‘accommodation’ step following initial formation of an ‘encounter complex’ stabilized by specific anticodon interactions. The proposed accommodation process involves conformational changes in both partners resulting in correct positioning of the CCA end in the active site (57).

In the case of ProXp-ala, which is significantly smaller than ProRS, the initial encounter does not involve anticodon interactions and both encounter and accommodation must rely exclusively on acceptor stem interactions. The proposed R80 side chain interaction with the major groove of G72 resembles the ProRS R144-G72 interaction reported earlier (56). We propose that the R80–G72 interaction contributes to the stabilization of the initial substrate encounter complex, with the K50 residue playing a more minor role. Our data also support previous data suggesting that a primary role for the nearly universally-conserved K45 residue among INS superfamily members is in the accommodation step; this residue is important for substrate binding (24), but is also proposed to steer the aminoacyl moiety into the active site via specific interactions with the phosphate between C75 and A76 (17,47,48,50,58). Our NMR data are consistent with a direct interaction between K45 and A76 and we additionally show that in the absence of the A76 2′-OH group, deacylation by *Cc* ProXp-ala is severely compromised. These data agree with an editing mechanism first proposed for the bacterial ProRS INS domain involving A76 2′-OH-mediated water activation (48).

Our new data together with previous results show that distinct sequence motifs encoded in ProRS and in the related ProXp-ala *trans*-editing domain both recognize the unique C1:G72 acceptor stem element. These results are reminiscent of an earlier study showing that the unique identity element for AlaRS, the G3:U70 base pair in the acceptor stem, is recognized by distinct domains: the aminoacylation and editing domains of AlaRS, and the related Ala-Xp *trans*-editing domain (59). A highly conserved Arg residue is also involved in G:U recognition and tRNA specificity in the tRNA^Ala^ editing systems (59,60). Thus, to avoid mistranslation errors that may have severe consequences for the cell, such as Pro to Ala substitutions that would be detrimental to protein structure, and Ala to Ser/Gly substitutions, which are known to result in severe neurodegeneration in mice (5), multiple checkpoints that rely on the same unique tRNA acceptor stem elements are used.

While bacterial tRNA^Pro^ encodes C1:G72, eukaryotic tRNA^Pro^ acceptor stems, including humans, encode G1:C72. This base pair together with C73 were recently shown to be critical for species-specific recognition of tRNA^Pro^ by human ProXp-ala (61). Future studies aimed at understanding the acceptor stem specificity of ProXp-ala encoded in human pathogens, as well as the distinct mode of acceptor stem recognition by the human enzyme may inform potential antibiotic development.

## DATA AVAILABILITY

The data underlying this article are available in the article and in its online supplementary material.

## Supplementary Material

gkad192_Supplemental_File
