# Supplementary material for: Structural basis of tRNAPro acceptor stem recognition by a bacterial trans-editing domain
Source: Nucleic Acids Res. 2023 Mar 23;51(8):3988–99. doi: 10.1093/nar/gkad192 (PMC10164551; doi:10.1093/nar/gkad192)
Supplement: gkad192_Supplemental_File [file gkad192_supplemental_file.pdf]

Supplementary Information for

Structural basis of tRNA^{Pro} acceptor stem recognition by a bacterial *trans*-editing domain

Xiao Ma[1], Marina Bakhtina[1], Irina Shugina[1], William A. Cantara[1], Alexandra B. Kuzmishin Nagy[1], Yuki Goto[2], Hiroaki Suga[2], Mark P. Foster[1,*], and Karin Musier-Forsyth[1,*]

[1] Department of Chemistry and Biochemistry and Center for RNA Biology, Ohio State University, Columbus, OH 43210, USA
[2] Department of Chemistry, Graduate School of Science, University of Tokyo, Bunkyo, Tokyo 113-0033, Japan
* To whom correspondence should be addressed. Tel: +1 614 292 2021; Fax: +1 614-688-5402; Email: musier-forsyth.1@osu.edu.
Correspondence may also be addressed to Mark P. Foster. Tel: +1 614 292 1377; Fax: +1 614 292 6773; Email: foster.281@osu.edu

This PDF file includes:
Supplementary Figure S1 to S4
Supplementary Table 1 to 3

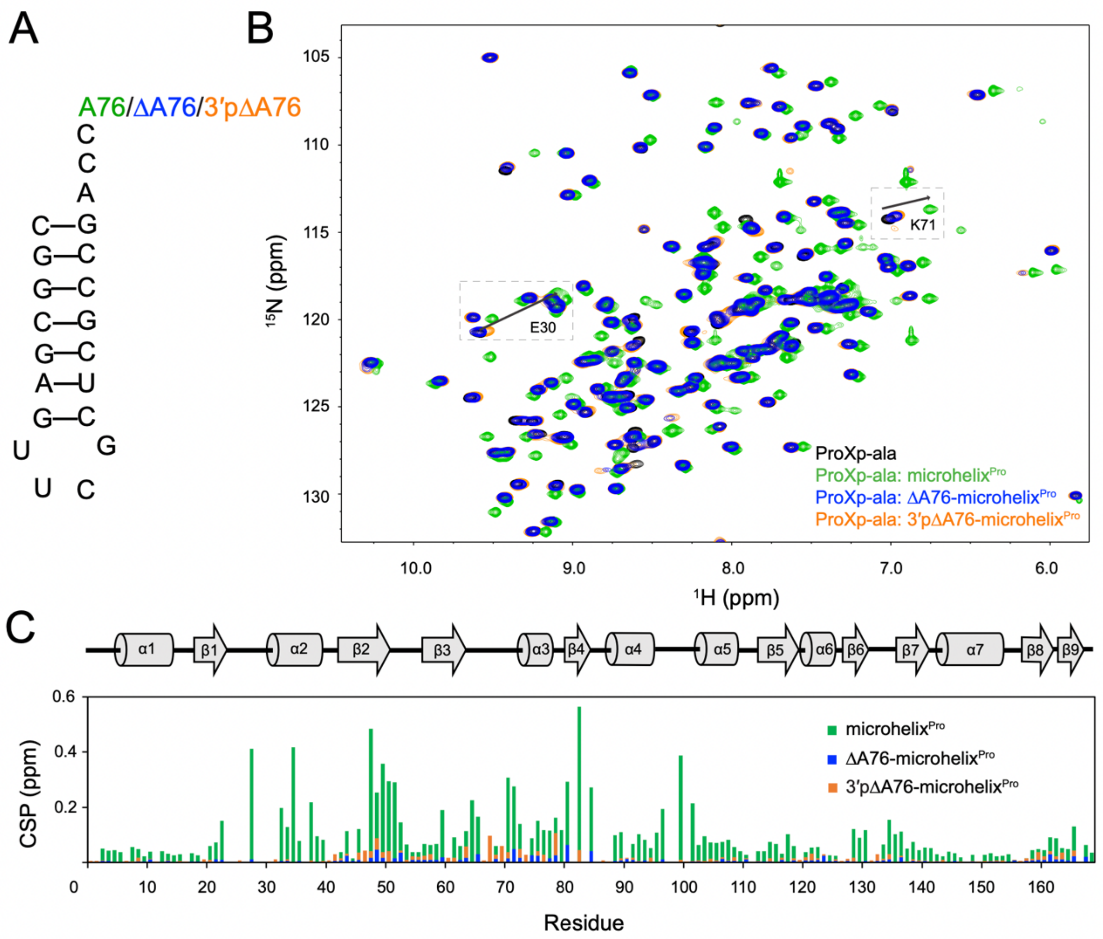

Supplementary Figure S1. Robust binding of tRNA^{Pro} ProXp-ala requires the 3′-A76 nucleotide. (A) Secondary structure of microhelix^{Pro} (green), ΔA76-microhelix^{Pro} (blue), and 3′pΔA76-microhelix^{Pro} (orange). (B) Overlaid ^{1}H-^{15}N HSQC spectra of [U-^{15}N]-ProXp-ala alone (black) and in the presence of microhelix^{Pro} (green), ΔA76-microhelix^{Pro} (blue), or 3′pΔA76-microhelix^{Pro} (orange); Boxes with dotted lines indicate spectral expansions in Figure 5B. (C) Secondary structure of *Cc* ProXp-ala (top) and summary of per residue CSPs induced in ProXp-ala by microhelix^{Pro} (green), ΔA76-microhelix^{Pro} (blue), and 3′pΔA76-microhelix^{Pro} (orange).

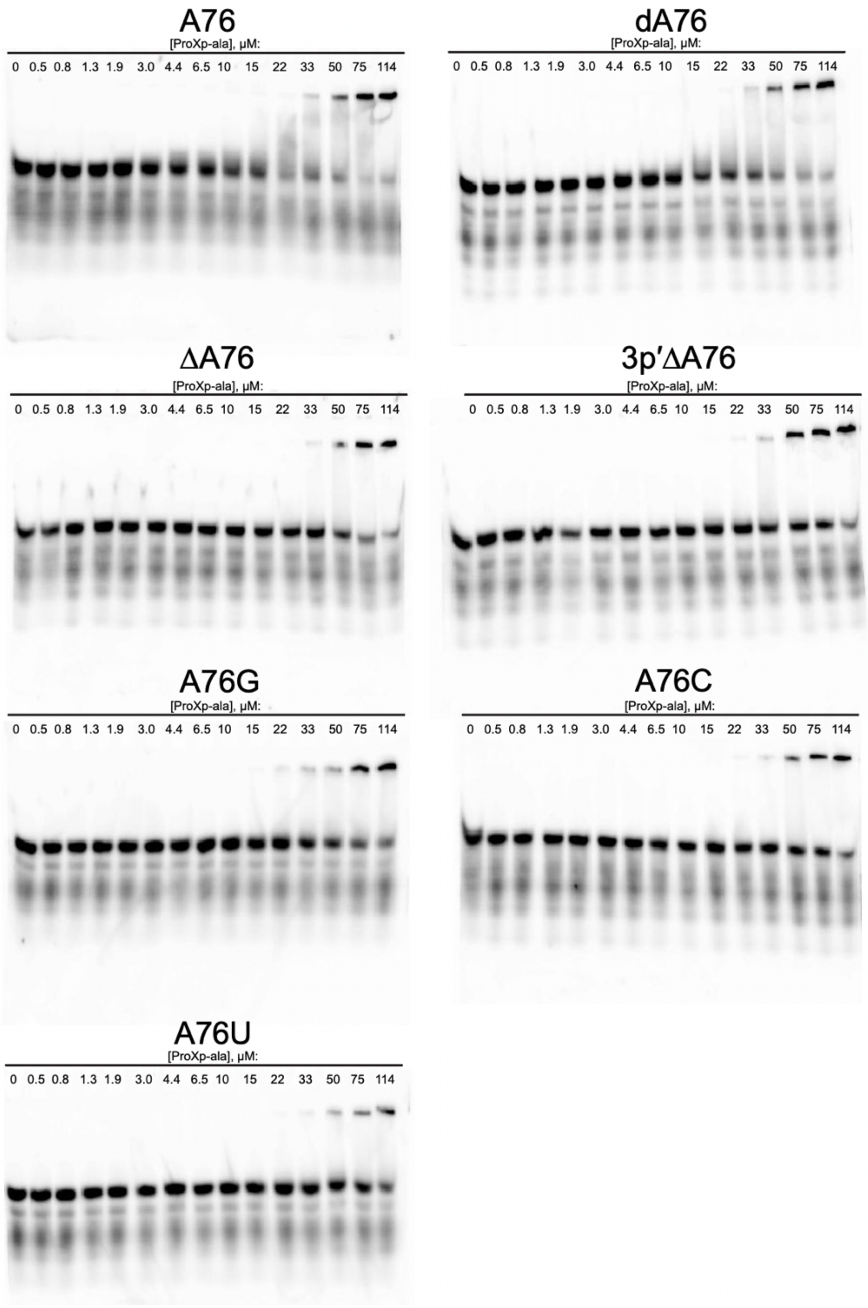

Supplementary Figure S2. Representative EMSA gels for the indicated duplex^{Pro} variants corresponding to data in Figure 5 and Table 1. In each case, 81 nM annealed RNA duplex was incubated with different amounts (0-114 μM) of ProXp-ala for 1 h before analysis on 16% native polyacrylamide gels. Due to the smeared bands, the complex formation was calculated from the disappearance of the free RNA, [1- (Free/Total RNA)]. Apparent equilibrium dissociation constants, K_D values, were obtained from fits to a hyperbolic equation using Excel Solver. Reported K_D values are the average of at least three independent experiments.

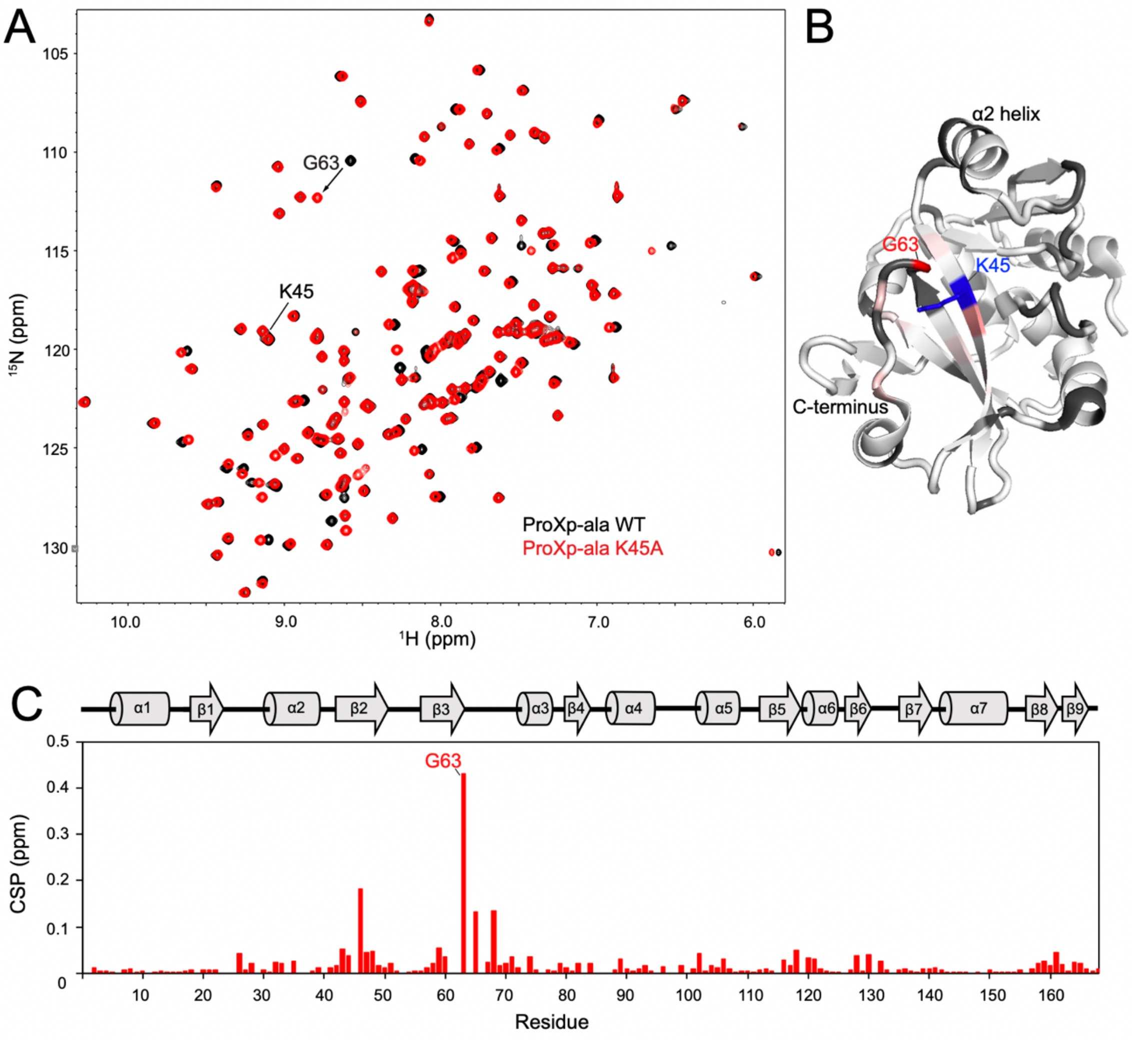

Supplementary Figure S3. NMR spectra show that poor substrate binding by K45A ProXp-ala is not due to a folding defect. (A) Overlaid ^{1}H-^{15}N HSQC spectra of [U-^{15}N]-ProXp-ala WT (black) and K45A variant (red). (B) Chemical shift differences between K45A and WT ProXp-ala mapped with a linear color gradient from white (no CSP) to red (CSP 0.43 ppm) on the crystal structure of *Cc* ProXp-ala (PDB: 5VXB). Unassigned residues are grey. K45 and G63 are labeled blue and red, respectively; (C) Secondary structure of *Cc* ProXp-ala (top) and per residue CSP analysis of K45A ProXp-ala compared to WT ProXp-ala.

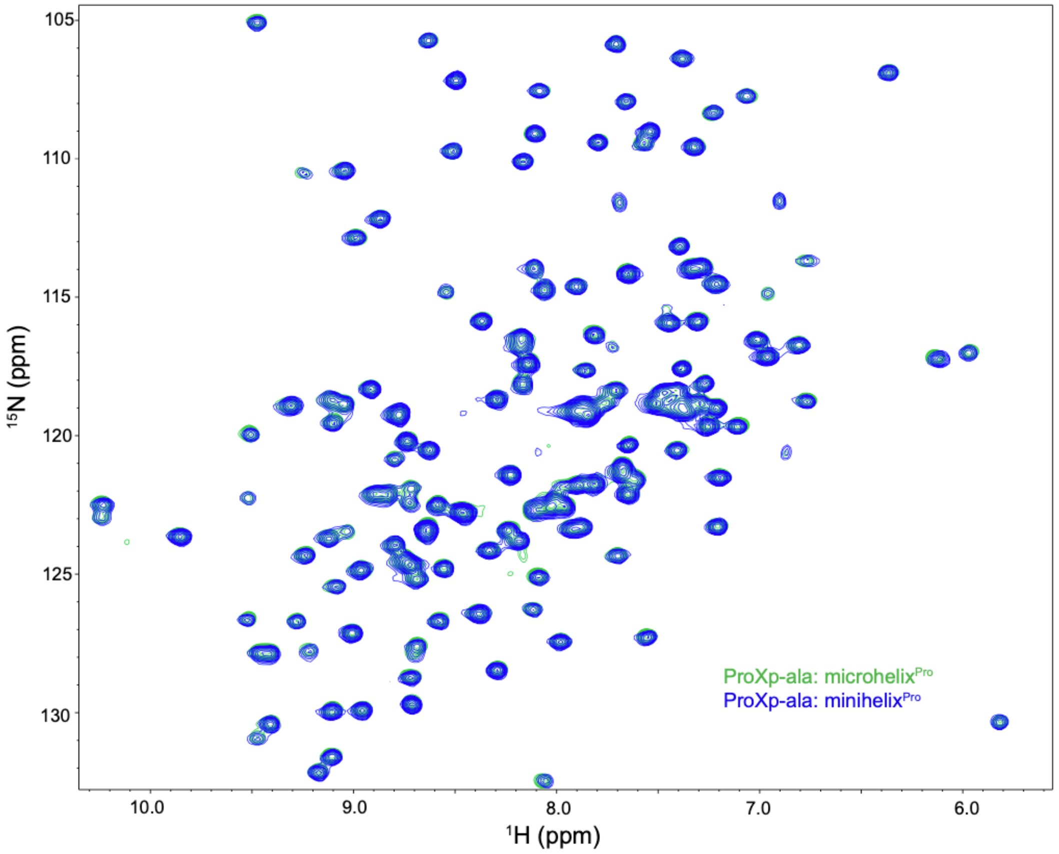

Supplementary Figure S4. Minihelix^{Pro} and microhelix^{Pro} bind similarly to ProXp-ala. Overlaid ^{1}H-^{15}N HSQC spectra of 100 μM [U-^{15}N]-ProXp-ala in the presence of 100 μM microhelix^{Pro} (green) or minihelix^{Pro} (blue). The spectra were recorded at 25 °C.

Supplementary Table 1. Sequences (shown 5′ to 3′) of the microhelix and minihelix RNA constructs used in this work.

RNA Constructs	Sequence
microhelix^{Pro}	CGGCGAGUUCGCUCGCCGACCA
G1:C72-microhelix^{Pro}	GGGCGAGUUCGCUCGCCCACCA
ΔA76-microhelix^{Pro}	CGGCGAGUUCGCUCGCCGACC
3′pΔA76-microhelix^{Pro}	CGGCGAGUUCGCUCGCCGAC[3′pC]
minihelix^{Pro}	CGGCGAGGGAGGUUCGAAUCCUCUCUCGCCGACCA

Supplementary Table 2. Sequences (shown 5′ to 3′) of the RNA duplex constructs used in this work.

5′ Fragment	Sequence
5′ fragment	CGGCGAGGGAGGUU[6FAM]
**3′ Fragments**	**Sequence**
A76 (WT)	AUCCUCUCUCGCCGACCA
ΔA76	AUCCUCUCUCGCCGACC
3′pΔA76	AUCCUCUCUCGCCGAC[3′pC]
A76C	AUCCUCUCUCGCCGACCC
A76G	AUCCUCUCUCGCCGACCG
A76U	AUCCUCUCUCGCCGACCU
dA76	AUCCUCUCUCGCCGACC[dA]

Supplementary Table 3. Results of double-exponential fits of deacylation data in Figures 2 and 3. The k_{obs} values plotted in Figure 3B were extracted from the fast phase, which on average represented at least 80% of the amplitude.

Trials	WT ProXp-ala and WT $tRNA^{Pro}$
1	$y = 87.3 \times e^{-23.4t} + 13 \times e^{-0.25t} - 0.3$
2	$y = 83.5 \times e^{-21.8t} + 14.6 \times e^{-0.35t} + 1.8$
3	$y = 82.7 \times e^{-21.6t} + 17.3 \times e^{-0.46t} + 0.04$
Trials	WT ProXp-ala and A73C $tRNA^{Pro}$
1	$y = 92.6 \times e^{-5.4t} + 7.3 \times e^{-0.14t} + 0.2$
2	$y = 85.5 \times e^{-6.9t} + 13.2 \times e^{-0.5t} + 1.2$
3	$y = 92.1 \times e^{-6.7t} + 7.8 \times e^{-0.36t} + 0.1$
Trials	K50A ProXp-ala and WT $tRNA^{Pro}$
1	$y = 79.2 \times e^{-5.4t} + 20 \times e^{-0.27t} + 0.8$
2	$y = 79.9 \times e^{-5.3t} + 19.6 \times e^{-0.27t} + 0.4$
3	$y = 82.1 \times e^{-4.6t} + 17.3 \times e^{-0.24t} + 0.6$
